# Lineage-Specific Changes in Biomarkers in Great Apes and Humans

**DOI:** 10.1371/journal.pone.0134548

**Published:** 2015-08-06

**Authors:** Claudius Ronke, Michael Dannemann, Michel Halbwax, Anne Fischer, Christin Helmschrodt, Mathias Brügel, Claudine André, Rebeca Atencia, Lawrence Mugisha, Markus Scholz, Uta Ceglarek, Joachim Thiery, Svante Pääbo, Kay Prüfer, Janet Kelso

**Affiliations:** 1 Institute of Laboratory Medicine, Clinical Chemistry and Molecular Diagnostics, University Hospital Leipzig, Leipzig, Germany; 2 Max Planck Institute for Evolutionary Anthropology, Leipzig, Germany; 3 Institute for Medical Informatics, Statistics and Epidemiology, University of Leipzig, Leipzig, Germany; 4 Lola Ya Bonobo Sanctuary, “Petites Chutes de la Lukaya,” Kinshasa, Democratic Republic of Congo; 5 Réserve Naturelle Sanctuaire à Chimpanzés de Tchimpounga, Jane Goodall Institute, Pointe-Noire, Republic of Congo; 6 Conservation & Ecosystem Health Alliance (CEHA), Kampala, Uganda; 7 College of Veterinary Medicine, Animal Resources & Biosecurity, Makerere University, Kampala, Uganda; Universitat Pompeu Fabra, SPAIN

## Abstract

Although human biomedical and physiological information is readily available, such information for great apes is limited. We analyzed clinical chemical biomarkers in serum samples from 277 wild- and captive-born great apes and from 312 healthy human volunteers as well as from 20 rhesus macaques. For each individual, we determined a maximum of 33 markers of heart, liver, kidney, thyroid and pancreas function, hemoglobin and lipid metabolism and one marker of inflammation. We identified biomarkers that show differences between humans and the great apes in their average level or activity. Using the rhesus macaques as an outgroup, we identified human-specific differences in the levels of bilirubin, cholinesterase and lactate dehydrogenase, and bonobo-specific differences in the level of apolipoprotein A-I. For the remaining twenty-nine biomarkers there was no evidence for lineage-specific differences. In fact, we find that many biomarkers show differences between individuals of the same species in different environments. Of the four lineage-specific biomarkers, only bilirubin showed no differences between wild- and captive-born great apes. We show that the major factor explaining the human-specific difference in bilirubin levels may be genetic. There are human-specific changes in the sequence of the promoter and the protein-coding sequence of uridine diphosphoglucuronosyltransferase 1 (UGT1A1), the enzyme that transforms bilirubin and toxic plant compounds into water-soluble, excretable metabolites. Experimental evidence that UGT1A1 is down-regulated in the human liver suggests that changes in the promoter may be responsible for the human-specific increase in bilirubin. We speculate that since cooking reduces toxic plant compounds, consumption of cooked foods, which is specific to humans, may have resulted in relaxed constraint on UGT1A1 which has in turn led to higher serum levels of bilirubin in humans.

## Introduction

Humans and their closest evolutionary relatives, the chimpanzees and bonobos, differ from one another in morphological, cognitive, behavioural and physiological traits [1–3]. The availability of genome sequences for human, chimpanzee, bonobo and rhesus macaque allows for the identification of lineage-specific sequence changes. However, since most of the sequence differences are likely to be neutral [4], it remains a challenging task to identify those that have important biological consequences [5]. For a relative small number of loci, human lineage-specific genomic changes have been linked to phenotypic differences to the other great apes [6]. One example is a deletion within the human gene *CMAH* which leads to the loss of the sialic acid Neu5Gc and to altered pathogen susceptibility [7].

Using the great apes to identify connections between genotype and phenotype in humans is hampered by the comparatively limited biomedical and physiological information about great apes [1, 5, 8]. Blood provides an opportunity to quantitatively analyze metabolic products that can serve as markers of metabolic and hormonal states [9]. The genes and metabolic pathways underlying these metabolic products are often well-characterized and it is therefore sometimes possible to identify the potential genetic basis for differences in metabolite levels.

While blood biomarkers are extensively studied in humans, there have been only limited comparative studies of humans and great apes. These that exist have been carried out in small numbers of captive animals [10, 11] or involve a single species [12–15].

**Fig 1:**
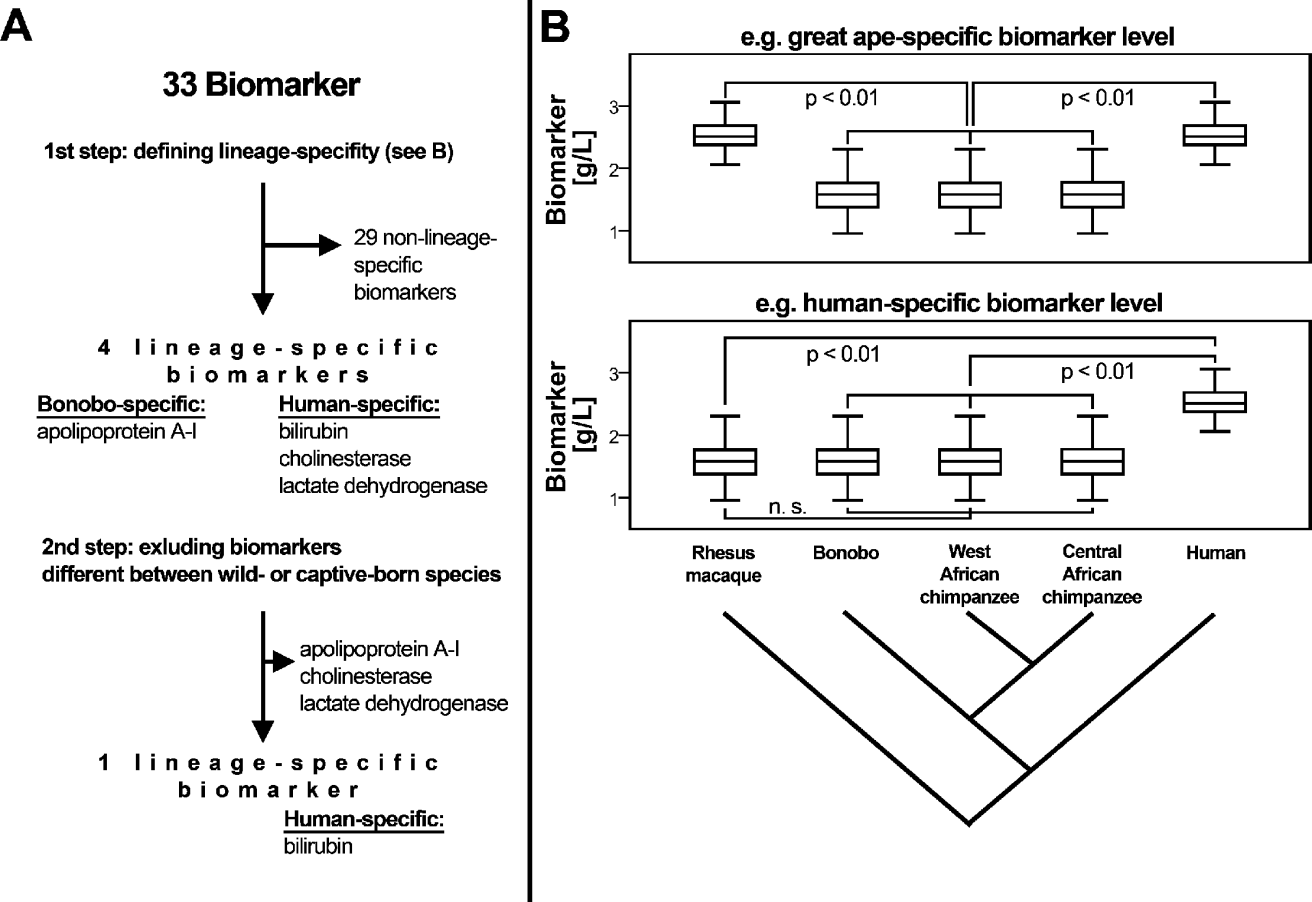
Flow chart of the analytic approach to identify lineage-specific biomarker levels. **A:** differences in biomarker levels were sorted as specific to (i) humans, (ii) great apes, (iii) bonobos, (iv) chimpanzees, (v) Central African chimpanzees, (vi) West African chimpanzees and as (vii) non-lineage specific. **B:** Human-specific changes were defined as significant differences to chimpanzees and bonobos taken together (but not between the latter two species) as well as to rhesus macaques (shown); and as significant differences between humans and the individual great ape species (not shown), regardless of significant differences between species born and living under different environments. Relations of species as shown in cladograms derived from [103, 104].

The aim of this study was to identify biomarkers that show lineage-specific changes in their levels or activity in the serum of humans, chimpanzees and bonobos. We analyzed serum samples from 277 wild-born and captive-born great apes (121 Central African chimpanzees, 95 West African chimpanzees, 61 bonobos), and from 312 healthy human volunteers from Germany. For each sample we measured up to 33 biomarkers that are routinely used in human and veterinary medicine to quantify heart, liver, kidney, thyroid and pancreas function, hemoglobin and lipid metabolism and one marker of inflammation. We assigned lineage-specific changes based on comparison to serum levels of the same biomarkers in 20 rhesus macaques from Germany (Fig 1). We identified biomarkers that are likely to have changed on one lineage and divide these into changes that are strongly influenced by environmental factors and changes that are unlikely to be explained by environmental effects alone.

## Methodology

### Ethics statement

All animal work was conducted according to relevant national, EU and international guidelines. In all cases, the animals were not subjected to any experimental procedures, and the blood samples used were left-over aliquots collected by veterinarians carrying out routine medical examinations. Authorization for use of the samples was obtained from the respective Ministries of Environment as well as by the Ministère de la Recherche Scientifique (DRC) to “Les Amis des Bonobos du Congo”, the Uganda Wildlife Authority and the Uganda National Council for Science and Technology, and the Ministère de l'Enseignement Supérieur et de la Recherche Scientifique from Republic of Congo. The international transport of samples was approved (CITES numbers: Uganda E-3520/05, Kenya E-1259/05, DRC E-0908/07, Republic of Congo E-1274/07). The proposal that in part covers this research (233297, TWOPAN) was reviewed and approved by the European Commission.

### Samples

Sera from 121 (65 male and 56 female) wild-born Central African chimpanzees (*Pan troglodytes troglodytes*), 76 (34 male and 42 female) wild-born and 19 (6 male and 13 female) captive-born West African chimpanzees (*Pan troglodytes verus*), 50 (28 male and 22 female) wild-born and 11 (7 male and 4 female) captive-born bonobos (*Pan paniscus*), 20 (3 male and 17 female) captive-born rhesus macaques (*Macaca mulatta*) and from 312 (156 male and 156 female) humans was used for this study.

All samples of wild-born great apes were collected in 2007 and 2009 during annually planned health checks. The samples of wild-born Central African chimpanzees were collected at Tchimpounga Sanctuary (Pointe Noire, Republic of Congo), the samples of wild-born West African chimpanzees were collected at Tacugama Sanctuary (Freetown, Sierra Leone) and the samples of wild-born bonobos were collected at Lola Ya Bonobo Sanctuary (Kinshasa, Democratic Republic of Congo).

The wild-born great apes were fasting from the evening before the anaesthesia and were then anaesthetized with a combination of medetomidine (Domitor, Pfizer, NY, USA, 0.03 to 0.05 mg/kg) and ketamine (Imalgen, Merial, GA, USA, 3 to 4 mg/kg), injected intramuscularly with either a dart (Telinject, France) or a syringe (Terumo, Japan). Blood was collected from the femoral vein with the Vacutainer (Becton Dickinson, NJ, USA) or with the Monovette-system (Sarstedt, Germany). Then blood was processed according to a standardized protocol in order to minimize preanalytic influences. Blood was allowed to clot for 30 minutes at room temperature, then centrifuged at 4° C for 10 minutes at 1600 g. The supernatant serum was transferred into a freezing tube, stored on dry ice, protected from light and transferred to our laboratory for subsequent biomarker analysis. Two bonobos were excluded from analysis since they died shortly after the sampling of the blood. One Central African chimpanzee was excluded since he was an outlier in a principle component analysis. Median ages for wild-born bonobos, Central and West African chimpanzees were 6 years (range: 3 to 20 years), 8 years (range: 2 to 42 years) and 4 years (range: 1 to 23 years), respectively (Table 1). Median weights were 18.8 kg (2.5^th^ to 97.5^th^ percentiles: 4.9 and 45 kg), 38.8 kg (2.5^th^ to 97.5^th^ percentiles: 10 and 67 kg) and 27 kg (2.5^th^ to 97.5^th^ percentiles: 9.9 and 53.2 kg).

**Table 1:** Results for clinical chemical biomarkers in serum samples from wild- and captive-born great apes (wild-born Central African chimpanzees from the Republic of Congo (Ch—CG), wild-born West African chimpanzees from Sierra Leone (Ch—SL) and captive-born chimpanzees from Germany (Ch—DE), wild-born bonobos from the Democratic Republic of the Congo (B—CD) and captive-born bonobos from Germany (B—DE)), captive-born rhesus macaques from Germany (Rh—DE), and from healthy human volunteers (H—DE).

		Species						
		Rh—DE	B—CD	B—DE	Ch—CG	Ch—SL	Ch—DE	H—DE
N *(m/f)*		20 *(3/17)*	50 *(28/22)*	11 *(7/4)*	121 *(65/56)*	76 *(34/42)*	19 *(6/13)*	312 *(156/156)*
Age [years]	*median*	3.5	6	14	8	4	20	27
	*range*	1–22	3–20	3–37	2–42	1–23	8–46	18–65
Alanine transaminase activity [μkat/L]	*median*	0.10	0.27	0.54	0.48	0.48	0.65	0.18
	*percentiles/range*	0.07–0.15	0.09–0.66	0.43–0.83	0.21–0.94	0.27–0.94	0.41–1.93	0.08–0.40
Albumin [g/L]	*median*	42.8	33.3	40.0	36.9	37.6	39.3	43.8
	*percentiles/range*	34.4–47.6	24.8–40.2	35.5–42.6	29.1–41.1	32.4–42.2	28.8–43.5	38.7–48.2
Apolipoprotein A [g/L]—bs	*median*	1.40	2.15	2.51	1.63	1.85	1.71	1.58
	*percentiles/range*	0.80–2.08	1.47–2.83	2.06–3.52	1.15–2.28	1.25–2.41	1.08–3.10	1.11–2.26
Apolipoprotein B [g/L]	*median*	0.37	0.79	0.76	0.59	0.70	0.73	0.72
	*percentiles/range*	0.20–0.47	0.50–1.33	0.48–0.97	0.35–0.89	0.46–0.92	0.29–1.24	0.42–1.31
Aspartate transaminase [μkat/L]	*median*	1.20	0.53	0.40	0.79	0.39	0.48	0.42
	*percentiles/range*	0.87–1.66	0.21–2.91	0.27–0.80	0.39–1.80	0.22–0.56	0.26–1.08	0.26–0.69
Total bilirubin^a^ [μmol/L]—hs	*median*	< 1.71	< 1.71	< 1.71	< 1.71	< 1.71	< 1.71	3.40
	*percentiles/range*	< 1.71	<1.71–2.70	< 1.71–2.00	< 1.71–2.40	< 1.71–2.40	< 1.71–2.10	< 1.71–13.34
Cholinesterase [μkat/L]—hs	*median*	201.2	156.9	214.0	199.7	208.0	209.3	126.0
	*percentiles/range*	136.9–448.9	98.0–282.0	159.60–339.90	105.6–345.2	124.8–280.1	154.8–364.9	72.0–191.2
Total cholesterol [mmol/L]	*median*	3.42	5.63	5.50	4.63	5.07	4.58	4.72
	*percentiles/range*	1.84–4.39	3.90–8.54	4.61–6.45	3.00–7.06	3.78–6.78	2.90–8.07	3.24–6.95
Colloid osmotic pressure [mmHg]	*median*	26.1	24.9	n. a.	22.4	22.2	n. a.	28.3
	*range*	22.3–29.2	20.5–29.3		19.0–25.4	18.7–24.1		24.2–32.5
C-reactive protein [mg/L]	*median*	1.11	23.90	0.84	2.32	0.53	1.00	0.72
	*percentiles/range*	< 0.30–27.09	0.71–239.24	< 0.30–3.39	1.00–45.80	< 0.30–50.82	< 0.30–3.57	< 0.30–10.28
Creatine kinase [μkat/L]	*median*	42.28	2.14	4.10	2.97	1.80	3.68	1.19
	*percentiles/range*	18.97–86.40	0.56–12.11	2.57–6.91	1.31–10.40	0.92–6.23	1.17–13.32	0.50–4.39
Creatinine [μmol/L]	*median*	76.0	46.5	82.0	62.0	58.5	75.00	70.0
	*percentiles/range*	52.0–118.0	14.5–93.9	34.0–123.0	33.1–121.9	35.6–103.1	50–253	48.5–95.0
Ferritin [ng/ml]	*median*	n. a.	21.3	71.5	105.4	69.1	203.2	27.0
	*percentiles*		4.0–157.2	31.0–171.9	15.5–388.2	18.0–268.8	17.5–883.3	5.5–144.6
Folate [nmol/L]	*median*	n. a.	31.50	21.18	28.31	18.69	36.16	20.56
	*percentiles*		15.43–45.40	12.84–45.40	17.51–45.40	11.02–29.84	28.50–45.00	9.27–39.36
Gamma-glutamyl transpeptidase [μkat/L]	*median*	1.59	0.10	0.15	0.26	0.32	0.44	0.28
	*percentiles/range*	0.72–3.40	< 0.05–0.32	0.09–0.34	0.11–0.99	0.18–0.58	0.18–1.13	0.12–1.75
Glucose [mmol/L]	*median*	n. a.	4.37	6.31	5.17	5.18	6.46	4.49
	*percentiles*		2.53–7.96	4.46–9.67	2.19–10.17	2.62–7.65	4.34–9.89	2.85–7.84
Glutamate dehydrogenase [μkat/L]	*median*	0.27	0.11	0.04	0.12	0.10	0.07	0.04
	*percentiles/range*	0.19–0.32	0.03–0.52	0.02–0.75	0.06–0.43	0.05–0.28	0.03–0.29	0.02–0.19
High-density lipoprotein cholesterol [mmol/L]	*median*	0.83	1.67	2.35	1.58	1.68	1.60	1.45
	*percentiles/range*	0.46–1.29	0.56–2.84	2.06–4.16	0.84–2.66	0.87–2.40	0.38–3.08	0.90–2.41
Lactate dehydrogenase [μkat/L]—hs	*median*	9.55	6.56	4.59	12.33	5.95	6.18	2.07
	*percentiles/range*	6.14–13.28	3.55–32.93	3.96–9.07	6.07–48.73	4.15–8.34	2.96–9.18	1.43–2.98
Lipase [μkat/L]	*median*	0.22	0.18	0.13	0.16	0.18	0.19	0.53
	*percentiles/range*	0.00–0.71	0.13–0.32	0.09–0.24	0.13–0.24	0.14–0.25	0.12–0.27	0.25–1.30
Lipoprotein (a) [mg/dl]	*median*	37.3	102.8	74.1	43.6	181.0	97.4	37.3
	*percentiles/range*	9.9–116.1	3.5–269.8	31.9–120.3	2.1–166.2	65.9–296.90	14.6–163.0	< 5.0–154.4
Low-density lipoprotein cholesterol [mmol/L]	*median*	1.89	3.03	2.90	2.48	2.66	2.40	2.72
	*percentiles/range*	0.42–2.50	1.71–5.94	1.79–3.47	1.29–4.06	1.67–4.03	1.71–5.64	1.41–4.60
N-terminal prohormone of brain natriuretic peptide [pg/mL]	*median*	<5.0	204.4	73.1	195.0	154.5	194.3	33.2
	*percentiles*	<5.0–9.9	26.9–2563.6	35.7–401.6	17.9–775.7	59.0–502.7	< 5.0–836.7	6.9–144.6
Phosphate [mmol/L]	*median*	n. a.	1.58	1.13	1.43	1.57	1.32	1.29
	*percentiles*		0.86–2.16	0.47–2.00	0.57–1.93	0.82–2.31	0.36–1.93	0.96–1.65
Total protein [g/L]	*median*	66.7	83.1	71.8	76.9	68.9	69.6	72.0
	*percentiles/range*	58.4–74.0	64.9–98.5	65.9–75.7	65.7–87.6	61.5–78.5	64.7–81.0	64.0–79.6
Thyroid-stimulating hormone [mU/L]	*median*	< 0.005	3.73	4.61	1.71	3.06	3.56	1.34
	*percentiles*	< 0.005–0.007	0.33–19.88	2.43–18.10	0.51–5.38	0.74–11.09	0.005–8.50	0.39–3.61
Free thyroxine [pmol/L]	*median*	14.14	11.33	14.41	12.37	12.14	16.61	16.53
	*percentiles/range*	7.92–24.37	6.53–18.37	10.26–25.19	7.19–20.87	8.15–21.03	11.40–24.21	13.11–20.76
Triglycerides [mmol/L]	*median*	0.69	1.15	0.56	1.02	1.12	1.08	1.28
	*percentiles/range*	0.44–1.01	0.58–3.11	0.28–1.70	0.56–2.30	0.63–1.94	0.73–3.06	0.55–3.95
Free triiodothyronine [pmol/L]	*median*	5.71	6.47	6.57	6.59	5.01	6.63	5.15
	*percentiles/range*	1.71–17.37	1.28–11.22	4.30–8.83	4.04–11.09	3.05–10.44	3.69–10.00	3.92–6.66
Troponin T [μg/L]	*median*	n. a.	< 0.01	< 0.01	< 0.01	< 0.01	< 0.01	< 0.01
	*percentiles/range*		< 0.01–0.18	< 0.01–0.04	< 0.01–0.05	-	< 0.01–0.25	< 0.01
Urea [mmol/L]	*median*	7.60	1.50	1.95	1.60	1.45	2.60	4.43
	*percentiles/range*	4.30–13.20	< 0.83–6.74	0.80–3.60	< 0.83–4.99	< 0.83–3.25	1.30–7.80	2.56–7.30
Uric acid [μmol/L]	*median*	< 11.9	203.0	128.0	145.5	145.5	124.0	265.5
	*percentiles*	< 11.9–12.0	106.4–371.6	93.0–192.0	75.0–226.8	64.5–221.8	81–224	142.1–407.5
Vitamin B12 [pmol/L]	*median*	n. a.	272.7	974.3	233.3	593.9	1476	278.9
	*percentiles/range*		126.8–1459.6	173.3 - >1476	82.4 - >1476	215.2 - >1476.0	1475 - >1476	147.5–576.6

ISO 3166 codes for the representation of countries of origin of serum samples; median, 2.5th—97.5th percentiles for wild-born great apes and humans and range for captive born great apes and rhesus macaques; ranges for age, colloid osmotic pressure and for lipoprotein (a) in wild-born Central African chimpanzees; “n. a.” = not analyzed; “bs” indicates a bonobo-specific biomarker and “hs” indicates human-specific biomarkers, green border denotes bonobo-specific biomarkers; blue border denotes human-specific biomarkers; “<”/”>” indicate below lower limit/above upper limit of quantification; percent of individuals below lower level of quantification (LLOQ) for bilirubin: wild- and captive-born bonobos: 82 and 55 percent, Central African chimpanzees: 75 percent; wild- and captive-born West African chimpanzees: 82 and 63 percent; rhesus macaques 100 percent; humans: 12.3 percent; see “Methodology” for details on the origin of the samples. The underlying data can be downloaded as S4 Dataset.

Samples from captive-born West African chimpanzees and bonobos were obtained from the Leipzig Zoo during routine health checks between 2005 and 2013. Animals fasted for 12 hours prior to anaesthesia with a combination of xylazine (2 to 3 mg/kg for bonobos and 2.4 to 4 mg/kg for chimpanzees) and ketamine (4 to 6 mg/kg for bonobos and 2.5 to 4 mg/kg for chimpanzees), injected intramuscularly with a dart. Blood was taken either from the vena saphena parva superficialis or from the vena mediana cubiti. Sera were stored at -80°C prior to analysis. No weights were available for captive-born great apes. Median ages for captive-born bonobos, and West African chimpanzees were 14 years (range: 3 to 17 years) and 20 years (range: 8 to 46 years), respectively (Table 1).

Sera from rhesus macaques were purchased from the German primate center (Göttingen/Germany). The sera were from healthy individuals kept for breeding purposes, and were collected during routine physical exams in May 2007 from the vena saphena of the proximal forth of the thigh. Animals had been anaesthetized with ketamine after a 12 hour fast. All sera were stored at -20° C prior to analysis. Although individual weights were not recorded for each rhesus macaque, males were between 2 and 3 kg and females between 7 and 8 kg. The median age for rhesus macaques was 3.5 years (range: 1 to 22 years; Table 1).

As the human reference group we used serum of a group of 312 young healthy blood donors previously described [16]. Median age was 27 years (range: 18 to 65 years; Table 1); median weight was 70 kg (2.5^th^ to 97.5^th^ percentiles: 52 and 98 kg).

### Biochemical analyses

All biochemical analyses were performed at the Institute of Laboratory Medicine, Clinical Chemistry and Molecular Diagnostics of the University Clinic Leipzig/Germany except for parts of the analysis of lipoprotein(a) (Lp(a)) which was performed at the Department of Medical Genetics, Molecular and Clinical Pharmacology, Divisions of Genetic Epidemiology and Human Genetics of the Innsbruck Medical University (Innsbruck/Austria).

Total cholesterol, HDL and LDL cholesterol, triglycerides, albumin, total protein, creatinine, urea and uric acid, bilirubin, glucose and phosphate were measured by using commercial photometric tests on an automated Modular P analyzer (Roche/Hitachi; S1 Dataset) [17–31]. Concentrations of apolipoprotein A-I and apolipoprotein B-100, C-reactive protein and Lipoprotein (a) were measured by using immunoturbidimetric assays (Tina-quant apo AI version 2 and Tina-quant apo B-100 version 2, C-reactive protein Gen.3; Tina-quant Lipoprotein (a), Roche; S1 Dataset) on Modular P [32–35]. The catalytic activities of alanine transaminase, aspartate transaminase, cholinesterase, creatine kinase, gamma-glutamyl transpeptidase, glutamate dehydrogenase, lactate dehydrogenase and lipase were determined using a photometric test on the same automated analyzer (S1 Dataset) [36–40]. Ferritin, free and total triiodothyronine and thyroxine, amino-terminal prohormone of brain natriuretic peptide (NTproBNP), troponin T and vitamin B12 were analyzed by a heterogeneous electrochemiluminescence immunoassay on an automated Modular E analyzer (Roche/Hitachi, S1 Dataset) [41, 42]. Lp(a) quantification in samples from the Republic of Congo, Sierra Leone and the Democratic Republic of Congo was performed as described in detail [43, 44] with a double-antibody enzyme-linked immunosorbent assay (ELISA), using an affinity-purified polyclonal apo(a) antibody for coating and the horseradish peroxidase-conjugated monoclonal antibody for detection. Colloid osmotic pressure was determined via direct hydrostatic pressure measurement on a colloid osmometer (Osmomat 50, Gonotec, Berlin, Germany) [45, 46] in triplicates.

A list of all biomarkers measured in this study with the corresponding method of detection, associated gene name (where possible) and pairwise alignment score for the comparison of proteins between humans and both West African chimpanzees and rhesus macaques is provided in S1 Dataset. A protein search was done using the HomoloGene function on NCBI’s webpage to obtain homologous protein sequences of human, West African chimpanzee and rhesus macaque proteins. When a homologous protein was not available for any of the non-human primate species using the HomoloGene function, a protein blast with the human protein was performed using UniProt to find the homologous protein in the remaining species.

**Fig 2:**
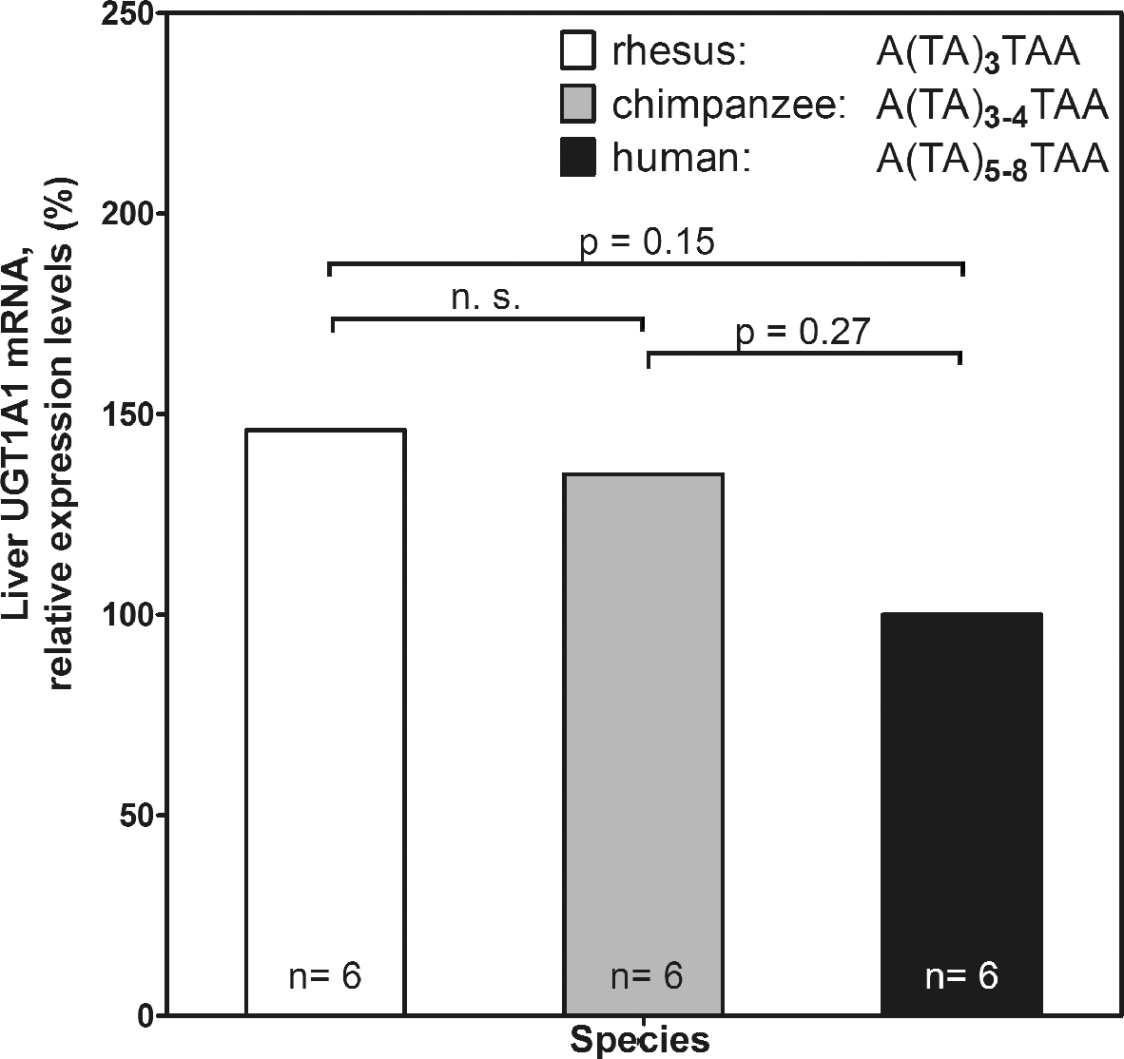
Liver UDP-glucuronosyltransferase 1A1 (UGT1A1) promoter transcript expression in rhesus macaques, chimpanzees and humans [47] and respective TATAA-box length [56, 57, 105] - UGT1A1 transcript expression was determined from RNA-Seq of human, chimpanzee and rhesus macaque liver RNA samples from 3 males and 3 females of each species [47]. Relative expression levels were calculated from the original dataset setting human expression levels at 100 percent (also see S1 Table for the variability of TA repeats in TATA box of UGT1A1 promoter in archaic hominins, humans and non-human primates).

The expression level of UGT1A1 transcripts in humans, chimpanzees, and rhesus macaques (Fig 2) was determined by transcript sequencing (RNA-Seq on an Illumina GA IIx) of liver samples from 3 males and 3 females from each species [47]. Significant differences in expression levels between species were determined using the negative binomial model implemented in DESeq [48].

**Fig 3:**
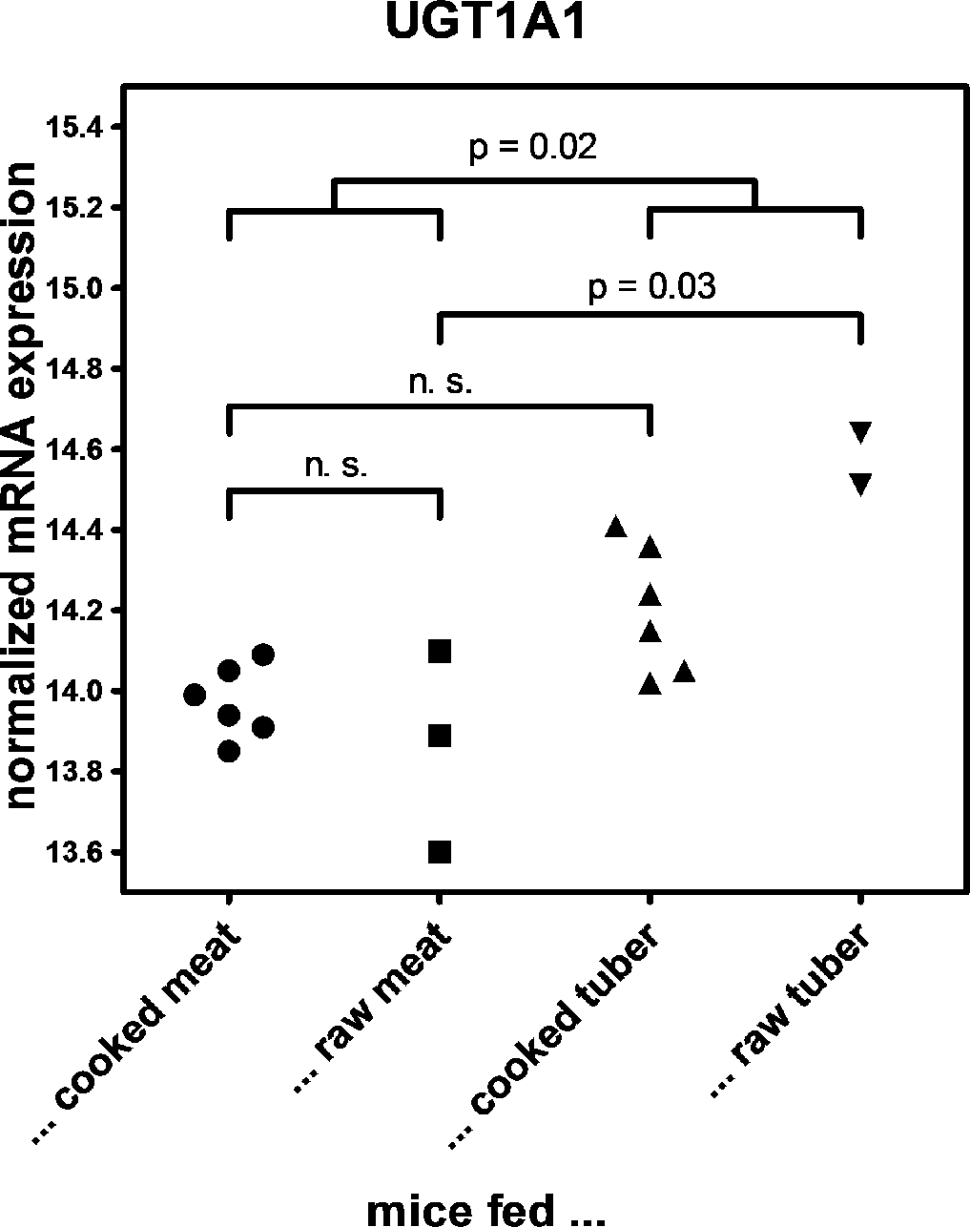
Liver *UGT1A1*-mRNA expression in mice on raw and cooked diets: Liver mRNA expression of UGT1A1 transcripts in mice fed either a raw or cooked meat or raw or cooked or tuber diets was measured by RNA-Seq [49]. Total RNA was prepared from 17 individuals and sequenced as a pool on two lanes of an Illumina HiSeq 2500. Significant differences in expression between mice fed raw diets and mice fed cooked diets were quantified using DESeq [48].

The expression levels of UGT1A1 transcripts in mice fed either a raw or a cooked diet composed of meat or of tuber were measured by RNA-Seq (Fig 3)[49]. Total RNA was prepared from 17 individuals and sequenced as a pool on two lanes of an Illumina HiSeq 2500. Significant differences in expression between mice fed raw diets and mice fed cooked diets were quantified using DESeq [48].

### Statistical analysis

Calculations were performed with IBM SPSS Statistics software (version 20.0.0) and R (version 2.12.1; http://cran.r-project.org/).

The study population was split into species groups. Percentages of change are given as medians (2.5th to 97.5th percentiles and ranges). The statistical significance of the differences between groups was assessed by using the Wilcoxon rank-sum test with the significance threshold set at 0.01.

## Results

### Sex-specific differences in serum biomarker levels

Our study included both male and female individuals and we therefore expect some differences in biomarker levels to reflect sex-specific traits. Using average timing of sexual maturity in each species [50] we expect that approximately 50 percent of the rhesus macaques, 30 and 73 percent of the wild- and captive-born bonobos, 49 percent of the wild-born Central African chimpanzees, 35 and 100 percent of the wild- and captive-born West African chimpanzees, and 100 percent of the humans in this study were sexually mature. We tested to what extent biomarker levels differ between males and females in each of the species. Sex-specific differences in serum biomarker levels are shown in S2 Dataset. Humans showed the largest number of significantly different biomarkers between females and males (67 percent of total number of biomarkers differed compared to between 0 and 12 percent in the non-human primates; S2 Dataset). In wild bonobos mean levels of low-density lipoprotein cholesterol, apolipoprotein A, apolipoprotein B and total cholesterol were significantly higher in females than in males. However, this difference was not seen in captive-born bonobos where levels of these same biomarkers were not significantly different between males and females (S2 Dataset).

Wild-born male Central African chimpanzees showed a significantly higher catalytic activity of creatine kinase, and higher levels of uric acid and ferritin than females. Cholinesterase was found to be significantly increased in wild-born male West African chimpanzees, while lactate dehydrogenase was significantly higher in captive-born West African male chimpanzees (S2 Dataset). In rhesus macaques no significant differences between the sexes were found for any of the biomarkers. In humans, all biomarkers with the exception of total cholesterol, colloid-osmotic pressure, folic acid, glucose, lactate dehydrogenase, lipoprotein (a), Thyroid-stimulating hormone, troponin T and vitamin B12 showed significant differences between the sexes (S2 Dataset).

### Lineage-specific biomarker levels

We assigned differences in biomarker levels as: (i) human-specific, (ii) great ape-specific, (iii) bonobo-specific, (iv) chimpanzee-specific, (v) Central African chimpanzee-specific, (vi) West African chimpanzee-specific and (vii) as uncategorized changes using the rhesus macaques as an outgroup (Fig 1). We did not identify rhesus macaque-specific changes since this would require an appropriate outgroup. Human-specific changes were defined as significant differences to all chimpanzees and bonobos taken together (but not between the two apes) as well as to rhesus macaques; and as significant differences between humans and the individual great ape species (Wilcoxon rank-sum test, p < 0.01). Using this approach, we identified human-specific differences in three biomarkers (bilirubin, cholinesterase, lactate dehydrogenase), and a bonobo-specific difference in one biomarker (apolipoprotein A). There was no biomarker difference specific to either the great apes as a group, to chimpanzees as a group or to West or Central African chimpanzees (Fig 1). Twenty-nine biomarkers showed differences that could not be assigned uniquely to one lineage because they were either not statistically significant or because they have changed on multiple lineages. These were classified as “non-lineage-specific”.

Amino acid differences in protein sequences may affect test results by altering antibody affinity for an analyte or the catalytic activities of enzymes. For three of the four biomarkers with species-specific differences, the genes underlying the biomarker can be identified. We compared the protein sequences of these genes between human, chimpanzee and rhesus macaque (S1 Dataset). The bonobo sequence of apolipoprotein A was 99.6, 99.6 and 94.8 percent identical to those from West African chimpanzees, humans and rhesus macaques, respectively. Human cholinesterase was 99.2, and 95.8 percent identical to those from West African chimpanzees, and rhesus macaques (S1 Dataset; a bonobo sequence was not available). Lactate dehydrogenase consists of three subunits (LDHA, LDHB and LDHC) with a median identity of 99.7 percent between humans and West African chimpanzees and a median identity of 98.9 percent between humans and rhesus macaques (S1 Dataset; a bonobo sequence was not available). As a metabolite, the fourth biomarker, bilirubin, is structurally identical in all species of our study [51].

### Biomarker level differences between wild- and captive-born individuals

Differences in biomarker levels may represent organismal responses to short- or long-term environmental factors, genetic differences between species, or both. We assume that biomarkers that show large differences between members of the same species living in different environments are those most affected by short-term environmental factors. We therefore compared each biomarker between wild- and captive-born individuals of the same species. We were only able to compare 32 biomarkers since colloid osmotic pressure was neither determined in captive-born bonobos nor in captive-born chimpanzees. Of these, 17 biomarkers differed between wild- and captive-born bonobos and 14 differed between wild- and captive-born chimpanzees; Wilcoxon rank-sum test, p < 0.01; S3 Dataset). When excluding the 25 biomarkers showing an environmental effect, only seven biomarkers remained (apolipoprotein B, bilirubin, total cholesterol, low-density lipoprotein cholesterol, N-terminal prohormone of brain natriuretic peptide, Thyroid-stimulating hormone and troponin T).

Biomarker levels that were significantly higher in both captive-born chimpanzees and captive-born bonobos were alanine transaminase, creatine kinase, ferritin, glucose, and vitamin B12 (S3 Dataset). The levels or catalytic activity of aspartate transaminase, gamma-glutamyl transpeptidase, the free thyroid hormones triiodothyronine and thyroxine, urea and folic acid were only significantly higher in captive-born chimpanzees but not in captive-born bonobos. The levels or catalytic activity of albumin, apolipoprotein A, cholinesterase and high-density lipoprotein cholesterol were only significantly higher in captive-born bonobos.

No biomarker was consistently higher in both wild-born great apes, whereas C-reactive protein, lactate dehydrogenase, lipase, total protein, triglycerides and uric acid were higher in wild-born and lipoprotein A and phosphate in wild-born chimpanzees (S3 Dataset).

### Quantification of influence of environment and species on biomarkers

To assess the relative influence of genetic and environmental factors in our dataset, we tested whether chimpanzees and bonobos from similar environments showed more similar biomarker levels than those from different environments. More specifically, we compared biomarker levels between captive- and wild-born bonobos and captive- and wild-born West African chimpanzees (see description of S1 Fig for details). We observe that wild-born bonobos and wild-born West African chimpanzees show the smallest differences. In contrast, comparisons between different environments (wild-born bonobos to captive-born West African chimpanzees and captive-born bonobos to wild-born West African chimpanzees) show significantly larger differences than the comparison between wild-born bonobos and wild-born chimpanzees (Wilcoxon rank-sum test, p < 0.001 in both comparisons; S1 Fig). Larger differences are also observed in the comparison of captive-born bonobos and chimpanzees. We conclude that short-term environmental influences may account for many of the observed differences in biomarker levels.

### Exclusion of biomarkers that are significantly different between individuals of the same species in different environments

To identify potential genetic differences for biomarker concentrations we eliminated from consideration biomarkers that differed between wild- and captive-born great apes since we reasoned that these are likely to be influenced by short-term environmental factors. Of the four lineage-specific biomarkers in our study, only bilirubin levels were equivalent among members of the same species from distinct habitats.

### A human-specific increase in serum bilirubin

**Fig 4:**
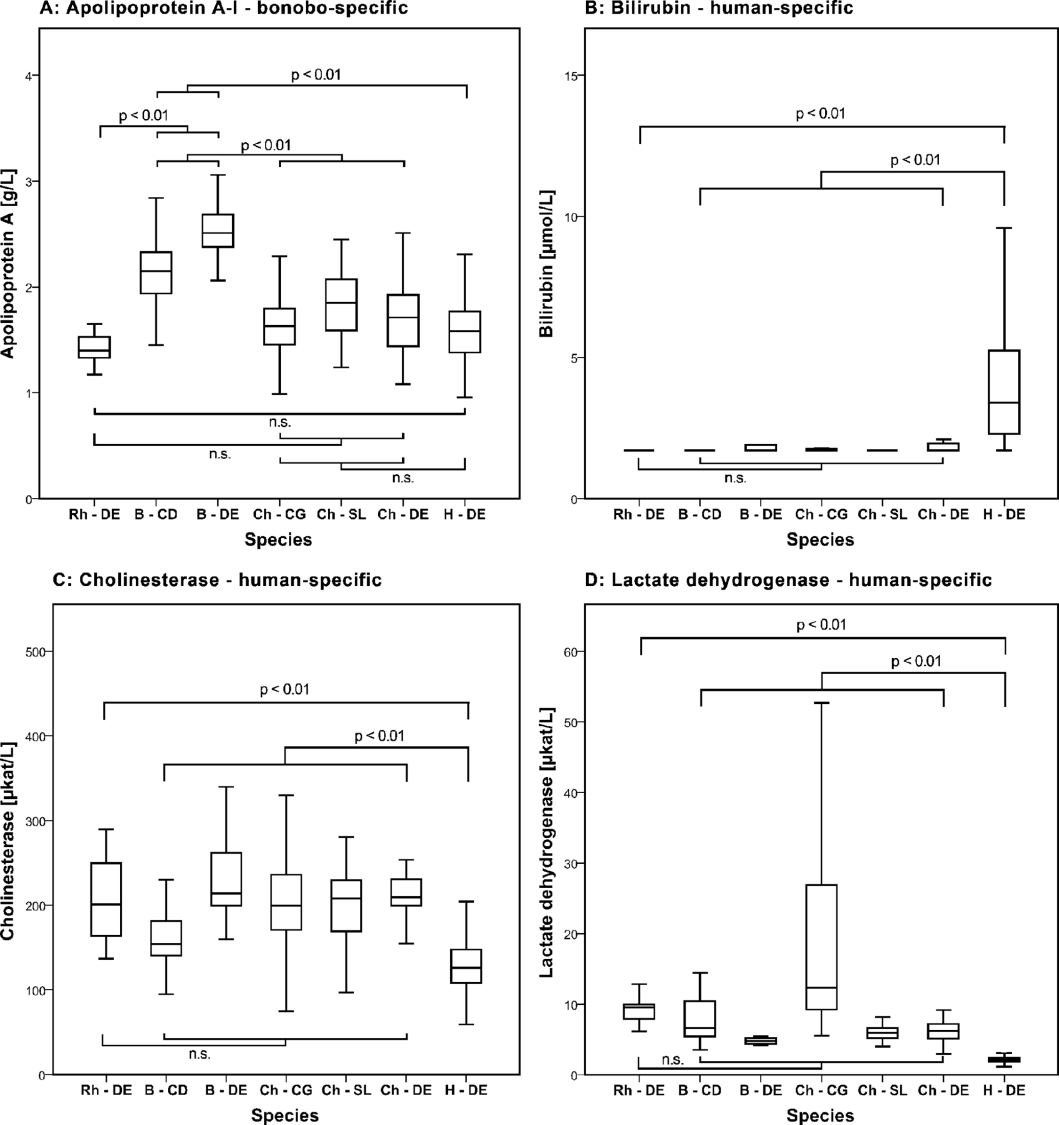
Lineage-specific biomarker levels in humans and other primates - box representing 25th, 50th and 75th percentiles; whiskers representing 2.5^th^ to 97.5^th^ percentiles; outliers are not shown; description of species: Rh—DE: captive-born rhesus macaque samples from Germany; B—CD: wild-born bonobo samples from the Democratic Republic of the Congo; B—DE: captive-born bonobo samples from Germany; Ch—CG: wild-born Central African chimpanzee samples from the Republic of Congo; Ch—SL: wild-born West African chimpanzee samples from Sierra Leone; Ch—DE: captive-born West African chimpanzee samples Germany; H—DE: human samples from Germany; A: Bonobo-specific change in apolipoprotein A-I; human-specific change in B: bilirubin, C: cholinesterase and D: lactate; for determination of bilirubin, 1.71 μmol/L represents the lower limit of quantification of the assay.

Humans have significantly higher serum levels of bilirubin than great apes and rhesus macaques (Median: 3.4 μmol/L in humans vs. < 1.71 μmol/L in the remaining species). While 12.3 percent of human samples were below the lower limit of quantification for bilirubin, the fraction in the other species was higher (between 55 and 82 percent in the great apes and 100 percent in the rhesus macaques) (Table 1; Fig 4).

Genome-wide association studies have shown that the major gene associated with serum bilirubin levels is *uridine diphosphoglucuronosyltransferase 1* (UGT1A1) [52–54]. The expression of *UGT1A1* largely depends on a microsatellite in the promoter of the *UGT1A1* gene, with an inverse relationship between the number of TA repeats and the activity of the gene [55]. The genome sequences show differences in TA repeat length with 3 repeats in rhesus macaques (reference assembly rheMac2), 3 to 4 repeats in chimpanzees (chimpanzee resequencing data from [56]) and 5–8 repeats in humans (13 human genomes from [57]; Fig 2, S1 Table). Furthermore, we found that the transcript expression levels of *UGT1A1* in rhesus macaque and chimpanzee livers were 46 and 35 percent higher than in humans (p = 0.15 and 0.27, respectively; Fig 2, S2 Table). The microsatellite length in two Neandertal individuals [57] and one Denisovan [58] are similar to present-day humans outside of Africa in having a TA repeat of length 6 in the promoter of *UGT1A1* (S2 Table).

By protein sequence alignment of *UGT1A1*, we found one human-specific amino acid substitution F518L (S2 Fig). The substitution lies between a transmembrane domain and di-lysine motifs in the cytosolic tail that confer retention of UGT1A-proteins to the endoplasmic reticulum (ER) and alter the half-life of UGT1A1 protein [59].

UGT1A1 is the main isozyme responsible for the glucuronidation of bilirubin. However, it is also involved in the glucuronidation that aides excretion of phytoalexins which are toxic plant-derived compounds [60]. To assess the effect of a plant- or animal-derived diet on *UGT1A1*-expression, we determined *UGT1A1* expression in liver samples of mice fed tuber or meat diets that were either cooked or raw (Carmody et al. in preparation). Mice on a raw tuber diet showed a tendency of higher *UGT1A1* expression than mice on either meat or cooked tuber diets (Fig 3).

## Discussion

The dataset presented here is, to our knowledge, the largest screen of biomarkers in wild- and captive-born great apes. While this dataset can be used for a variety of analyses, we have chosen to concentrate on the identification of lineage-specific changes in biomarker levels.

Large effort has been made to minimize preanalytic influences. All samples, including those from Africa, were frozen immediately after serum extraction. Studies have shown that long-term storage (up to 10 years) does not affect the results for any of the biomarkers we have tested [61]. All analyses were carried out using the same automated analyser.

We note that all great apes had to be sedated before sampling. Ketamine anaesthesia has been shown to influence blood levels of some of the biomarkers we measured (alanine and aspartate aminotransferase and creatine kinase [62, 63], phosphate [64]) whereas bilirubin is not changed [65, 66]. However, the differences we observe between captive-born and wild-born apes indicate that anaesthesia is not a major factor determining differences in blood-chemistry between groups.

All animals included in this study were deemed healthy by a trained veterinarian. However, we cannot exclude that subclinical infection could explain high values of C-reactive protein in the bonobos from Lola Ya Bonobo.

Our laboratory analyses were performed using established automated assays optimized for use in human clinical medicine. We are aware that this may affect results obtained in other species [10, 11, 67]. The sequence identity of proteins measured in our study lies between 95.1 and 100 percent compared to humans for West African chimpanzees and between 87.2 and 100 percent for rhesus macaques (S1 Dataset). Results obtained for some antibody-based assays (e.g. immunoassays) may therefore be species-dependent. Furthermore, serum catalytic activity as the product of enzymatic concentration and activity of each enzyme may be influenced by differences in protein sequence and/or structure.

Our study included serum samples from both male and female individuals. As sex-specific biomarker traits may affect overall biomarker level distributions, we analyzed our data for gender-specific differences. The number of significantly different biomarkers between sexes was greatest in humans compared to all other species (S2 Dataset). The reason for this finding is not clear. The finding is not consistent with the extent of sexual dimorphism [68]. Possible explanations include differences in proportions of male and female individuals in our study groups (female excess among rhesus macaques and captive-born chimpanzees, Table 1) and the proportion of mature individuals (highest in our human study group).

We find evidence for only few species-specific biomarker differences. Lactate dehydrogenase, cholinesterase and bilirubin showed human-specific differences and apolipoprotein A showed a bonobo-specific difference.

We found many differences in biomarker levels between members of the same species born and living under different environments. The biomarker levels that showed significant differences between captive- and wild-born individuals include those that are known to be influenced by dietary factors (ferritin, folic acid, glucose, the free thyroid hormones triiodothyronine and thyroxine, vitamin B12) and enzymes whose release into blood is dependent upon muscle mass or activity (aspartate transaminase, creatine kinase; S3 Dataset and Table 1) [26]. Except for bilirubin the comparison between captive-born and wild-born apes indicates that environmental differences may explain these lineage-specific differences.

In our study levels of apolipoprotein A-I were specifically higher in bonobos. In agreement with this result, levels of apolipoprotein A-I and high-density lipoprotein cholesterol have been found to be similar in captive-born West African chimpanzees and humans [10]. However, a study comparing lipid status in free-ranging and captive macaque species and humans found that levels varied more between the same species in different environments than between the different macaque species [67]. Further, apolipoprotein A-I was found to be elevated in female wild-born bonobos, female captive-born chimpanzees, and in female humans whereas this pattern was not seen in the remaining species. High-density lipoprotein cholesterol levels, which are highly correlated with apolipoprotein A-I, are known to be influenced by both diet and exercise in both humans and macaques [19, 26, 69–73]. We therefore cannot exclude that the lineage-specific change in bonobos is due to some environmental factor.

Lactate dehydrogenase showed a human-specific decrease compared to the apes and rhesus macaques. However, we detected significant variation in serum LDH activity between wild- and captive-born great apes, suggesting that LDH activity is likely to be environmentally influenced. As a cytoplasmic enzyme and constituent of all tissues, lactate dehydrogenase is liberated into the blood in response to tissue damage, which may also include muscle activity and alarm reactions before blood taking [26]. It is therefore conceivable that the non-human primates in our study group have higher lactate dehydrogenase activity due to a difference in physical activity.

Serum cholinesterase activity showed a human-specific decrease. The exact function of serum cholinesterase is unclear [74]. It is abundant in plasma, liver, pancreas, spleen and the white matter of the brain and is commonly used in clinical medicine to quantify liver synthetic capacity [26]. In humans genetic variants with catalytic activities that differ by a factor of ten exist [26]. Cholinesterase activity is furthermore known to be associated with weight, body mass index (BMI) and muscle mass [75]. Possible explanations for the observed differences may include a combination of the above mentioned factors.

The protein sequences of apolipoprotein A-I, serum cholinesterase and lactate dehydrogenase differ between species (S1 Dataset). It is not known whether these differences change their interaction with binding antibodies or their catalytic activity and therefore alter the measurement of these biomarkers with the standard assays applied here. For example, in our study apolipoprotein A-I was determined with an immunoturbidimetric assay that may be sensitive to protein differences. The human and chimpanzee apolipoprotein A-I protein sequence is identical while bonobos differ from both by one amino acid. We can therefore not distinguish whether the lineage-specific changes in apolipoprotein A-I in bonobos, and serum cholinesterase and lactate dehydrogenase in humans are due to differences in their protein sequence (S1 Dataset).

Bilirubin levels were consistently higher in humans than in West and Central African chimpanzees and bonobos (both captive and wild-born), as well as in rhesus macaques. This is in agreement with a small study that showed that bilirubin levels are twice as high in humans as in captive chimpanzees [10].

Serum concentration of bilirubin is a function of extrahepatic formation and intrahepatic excretion of bilirubin. The magnitude of bilirubin formation depends mainly on erythrocyte half-life [76, 77]. Since the erythrocytes of chimpanzees and rhesus macaques have a half-life which is about 50 percent shorter than that of human erythrocytes, bilirubin production should be higher in the great apes and rhesus macaques [78, 79]. However, we observe serum bilirubin levels that are lower in all the non-human primates, suggesting that bilirubin depletion and not bilirubin formation may cause the difference in levels.

To explore whether genetic changes might explain this difference we focused on the gene encoding UDP-glycosyltransferase 1 (*UGT1A1*) which is the major enzyme involved in the glucuronidation of bilirubin (99.5 percent of total turnover) and in the excretion of phytoalexins which are toxic plant-derived compounds [60, 80, 81]. Levels of bilirubin are highly heritable and association studies have shown that *UGT1A1* is the main contributor to these levels [52, 53]. The gene has not been found to vary in copy number [82]. However, Gilbert’s syndrome (GS, MIM*143500), an inherited form of mild hyperbilirubinemia, is typically caused by a homozygous TA insertion in the TATAA element of the 5’ promoter region in Europeans (normal A(TA)_6_TAA) (UGT1A1*28) [83]. Homozygotes for the A(TA)_7_TAA sequence have higher levels of bilirubin whereas bilirubin levels of heterozygotes are between homozygotes and subjects carrying the wild type [83, 84].

While the promoter repeat length varies from 5 to 8 in humans, rhesus macaques and chimpanzees show a reduced repeat length of 3 and 3 to 4, respectively. Based on the shorter promoter repeat length we would predict that UGT1A1 expression is reduced in humans compared with non-human primates. Indeed we find RNA expression to be 46 and 35 percent higher in rhesus macaques and chimpanzees than in humans (Fig 3. And S2 Table). Notably, the *UGT1A1* promoter in the Neandertal and Denisovan genomes (S1 Table) carries the sequence A(TA)_6_TAA, common in humans today. Since the Michaelis constant K_m_ of UDP-glycosyltransferase (the substrate concentration at which the reaction rate is half of the maximum rate) lies at bilirubin levels of 0.26 μmol/l [85], well below the median bilirubin of 3.4 μmol/l in humans, lower expression of *UGT1A1* may well increase serum bilirubin levels.

Protein sequence alignment of *UGT1A1* revealed one human-specific amino acid substitution at position F518L (S2 Fig). Interestingly, this substitution is situated between di-lysine motifs in the cytosolic tail and a transmembrane domain that confer retention of UGT1A-proteins to the endoplasmic reticulum (ER) [59]. This amino acid substitution between two ER-retention signals may alter the percentage of protein that stays in the ER and thus the turnover of UGT1A1 independently of expression differences. Further work is necessary to elucidate if and to what extent this protein difference and the promoter difference contribute to the altered bilirubin concentrations in humans.

In the process of heme degradation, mammals reduce water-soluble biliverdin to the potentially toxic [86–91] bilirubin in an energy-dependant step [51]. Because bilirubin is poorly soluble in water, it is further conjugated with glucuronic acid by UGT1A1 and transported into the bile, both steps requiring additional energy. Whether this particular mode of heme catabolism serves further purposes remains elusive [92]. Both *in vitro* [51, 93] and *in vivo* [94, 95] anti-oxidative and neuroprotective [96] properties of bilirubin have been known for many years. Clinical studies have shown associations between elevated bilirubin levels and lower incidence of cardiovascular disease [97], respiratory disease [98], cancer [99] and stroke [100]. However, it is not clear if this association is due to the inherently higher bilirubin levels or whether more bilirubin is degraded due to the disease [92].

Modern human populations derive more of their dietary energy from meat than the great apes (Leonard, Snodgrass et al. 2007). Improved diet quality due to cooking and consumption of animal protein and fat has been proposed as one among other factors allowing the increased brain size in humans [101, 102]. Our results indicate that expression of *UGT1A1* is higher in mice fed a raw plant diet than in mice fed either a meat or cooked-plant diet (Fig 3), consistent with the role UGT1A1 plays in the detoxification and excretion of plant toxins [60]. We hypothesise that the consumption of cooked food in the last million years of hominin history may either have reduced the selective constraint on sufficiently high levels of UGT1A1 expression or even driven reduction in the levels of UGT1A1 expression. The higher level of bilirubin may be a side-effect of the change in UGT1A1 expression, but may also have come under selection on its own right due to the beneficial effects associated with higher levels of bilirubin. The change in bilirubin may thus constitute an example of the wide-ranging consequences of cooking in the evolution of humans.

## Supporting Information

S1 Dataset Alignment scores. Alignment scores were derived from the NCBI and UniProt websites.
(XLSX)

S2 Dataset Influence of sex on biomarker measurements. (XLSX)

S3 Dataset Influence of environment on biomarker measurements using wild- vs. captive-born great apes. (XLSX)

S4 Dataset Supplementary dataset to lineage-specific changes in biomarkers in great apes and humans. (XLSX)

S1 Fig Divergence of biomarker levels in various great ape species. Distributions of p-values in all pairwise comparisons of 8 individuals to 8 individuals between two groups. Brackets on top and bottom show the results of a two-sided Wilcoxon rank test between distributions that are informative for environmental effect (zoo vs. wild compared to either zoo vs. zoo or wild vs. wild).
(DOCX)

S2 Fig Alignment of UDP-glucuronosyltransferase (UGT) 1A1 protein in humans and non-human primates. Red circle indicating human-specific amino acid substitution (F518L); sequences derived from [56, 106–108]; descriptions of protein folding and functional domains [59, 109–111].
(DOCX)

S1 Table Variability of TA repeats in TATA box of UDP-glucuronosyltransferase (UGT) 1A1 promoter in archaic hominins, humans and non-human primates. Sequences derived from [56–58, 82, 84, 105, 112, 113].
(DOCX)

S2 Table Expression data of genes analyzed in this study. Data derived from [47, 114, 115].
(DOCX)
