# Supplementary material for: Lineage-Specific Changes in Biomarkers in Great Apes and Humans
Source: PLoS One. 2015 Aug 6;10(8):e0134548. doi: 10.1371/journal.pone.0134548 (PMC4527672; doi:10.1371/journal.pone.0134548)
Supplement: S1 Fig — Distributions of p-values in all pairwise comparisons of 8 individuals to 8 individuals between two groups. Brackets on top and bottom show the results of a two-sided Wilcoxon rank test between distributions that are informative for environmental effect (zoo vs. wild compared to either zoo vs. zoo or wild vs. wild). (DOCX) [file pone.0134548.s005.docx]

**S1 Figure:**

**Divergence of biomarker levels in various great ape species**

*Introduction*

Differences in the levels of biomarkers can be caused by either genetic differences or by differences in environmental factors. In order to test whether the environment contributes to our results, we compared the levels of 31 biomarkers in captive- and wild-born bonobos to the levels in captive- and wild-born western chimpanzees (colloid osmotic pressure and troponin T were excluded since these measurements were not available in zoo individuals and in wild western chimpanzee, respectively). We then tested whether chimpanzees and bonobos from similar environments showed more similar biomarker levels.

*Method*

Different numbers of captive- and wild-born individuals were measured for each species. In order to equalize statistical power in all comparisons, we subsampled wild western chimpanzees, wild bonobos and zoo western chimpanzee down to 8 individuals (the number of zoo bonobos individuals with measurements for all biomarkers). 100 random draws of unique combinations of 8 individuals were performed for wild-born western chimpanzee. Since only 10 wild-born bonobos and 11 captive western chimpanzees had measurements for all 31 biomarkers, all possible unique permutations of 8 individuals were drawn for these groups.

In each comparison of 8 individuals to 8 individuals, we combined the information of the 31 biomarkers in order to increase power. For each biomarker, all 16 individuals are sorted according to their value and the rank was assigned to each individual. Ties in values were assigned to the mean ranks over the tie. The ranks of all biomarkers were then combined and a two-sided Wilcoxon rank test was applied to test for a significant difference in ranks between the groups.

*Results*

S1 Figure shows the distributions of p-values of the Wilcoxon rank test for all pairwise comparisons of 8 individuals to 8 individuals between groups. Large differences are observed in the comparison of wild to captive individuals, as expected if environmental effects contribute more to these comparisons than comparisons between groups of wild individuals. However, large differences are also observed in the comparison of zoo individuals. We conclude that environmental effects contribute to our results and have excluded biomarkers from further discussion that show significant differences between wild and captive-born individuals (see main text for details).

**S1 Figure:** Distributions of p-values in all pairwise comparisons of 8 individuals to 8 individuals between two groups. 100 random draws of 8 individuals were performed for wild-born western chimpanzee, while all unique combinations of 8 individuals were generated for the remaining groups. Brackets on top and bottom show the results of a two-sided Wilcoxon rank test between distributions that are informative for environmental effect (zoo vs. wild compared to either zoo vs. zoo or wild vs. wild).
