# Supplementary material for: Lineage-Specific Changes in Biomarkers in Great Apes and Humans
Source: PLoS One. 2015 Aug 6;10(8):e0134548. doi: 10.1371/journal.pone.0134548 (PMC4527672; doi:10.1371/journal.pone.0134548)
Supplement: S2 Fig — Red circle indicating human-specific amino acid substitution (F518L); sequences derived from [56, 106–108]; descriptions of protein folding and functional domains [59, 109–111]. (DOCX) [file pone.0134548.s006.docx]

**S2 Figure:**

**Alignment of UDP-glucuronosyltransferase (UGT) 1A1 protein in humans and non-human primates**

To evaluate human-specific changes in the UGT1A1 protein, we aligned the human UGT1A1 protein with homologous proteins from chimpanzee, bonobo, orangutan and rhesus macaque. The human amino acid sequence of UGT1A1 was derived from Uniprot (P22309), the bonobo variant from our own data (CCDS2510.1_bonobo) [[1](#_ENREF_1)] and the remaining sequences from the CCDS database (CCDS2510.1_chimp; CCDS2510.1_orang; CCDS2510.1_rhesus) [[2-5](#_ENREF_2)]. Descriptions for protein folding and functional domains are based on a published model by Laakkonen and Finel [[6](#_ENREF_6)]. Carboxy-terminal di-lysine motifs with endoplasmic reticulum retention signals based on publications from Pääbo et al.[[7](#_ENREF_7)] and Teasdale et al.[[8](#_ENREF_8)]. Additionally, the transmembrane domain confers static retention that prevents UGT1A1 exiting the endoplasmic reticulum [[9](#_ENREF_9)]. The only human-specific amino-acid substitution in the comparison of the five species is located at position 518 at the border of the transmembrane domains in the cytoplasmic tail with its dilysine motifs. The substitution changes a phenylalanine (F) present in all great apes into a leucine (L) in humans. Neither the Ensembl annotation nor Swiss-Prot have this variant annotated as being disease-associated.

**S2 Figure: Alignment of UDP-glucuronosyltransferase (UGT) 1A1 protein in humans and non-human primates -**  red circle indicating human-specific amino acid substitution (F518L); sequences derived from [[1](#_ENREF_1),[3-5](#_ENREF_3)]; descriptions of protein folding and functional domains [[6-9](#_ENREF_6)]

1. Prüfer K, Munch K, Hellmann I, Akagi K, Miller JR, et al. (2012) The bonobo genome compared with the chimpanzee and human genomes. Nature.

2. Consortium TU (2014) Activities at the Universal Protein Resource (UniProt). Nucleic Acids Research 42: D191-D198.

3. Farrell CM, O'Leary NA, Harte RA, Loveland JE, Wilming LG, et al. (2014) Current status and new features of the Consensus Coding Sequence database. Nucleic Acids Res 42: D865-872.

4. Harte RA, Farrell CM, Loveland JE, Suner MM, Wilming L, et al. (2012) Tracking and coordinating an international curation effort for the CCDS Project. Database (Oxford) 2012: bas008.

5. Pruitt KD, Harrow J, Harte RA, Wallin C, Diekhans M, et al. (2009) The consensus coding sequence (CCDS) project: Identifying a common protein-coding gene set for the human and mouse genomes. Genome Res 19: 1316-1323.

6. Laakkonen L, Finel M (2010) A molecular model of the human UDP-glucuronosyltransferase 1A1, its membrane orientation, and the interactions between different parts of the enzyme. Molecular Pharmacology 77: 931-939.

7. Pääbo S, Bhat BM, Wold WS, Peterson PA (1987) A short sequence in the COOH-terminus makes an adenovirus membrane glycoprotein a resident of the endoplasmic reticulum. Cell 50: 311-317.

8. Teasdale RD, Jackson MR (1996) Signal-mediated sorting of membrane proteins between the endoplasmic reticulum and the golgi apparatus. Annual review of cell and developmental biology 12: 27-54.

9. Barré L, Magdalou J, Netter P, Fournel‐Gigleux S, Ouzzine M (2005) The stop transfer sequence of the human UDP‐glucuronosyltransferase 1A determines localization to the endoplasmic reticulum by both static retention and retrieval mechanisms. FEBS Journal 272: 1063-1071.
