# Supplementary material for: Lineage-Specific Changes in Biomarkers in Great Apes and Humans
Source: PLoS One. 2015 Aug 6;10(8):e0134548. doi: 10.1371/journal.pone.0134548 (PMC4527672; doi:10.1371/journal.pone.0134548)
Supplement: S1 Table — Sequences derived from [56–58, 82, 84, 105, 112, 113]. (DOCX) [file pone.0134548.s007.docx]

**Table S1:**

**Variability of TA repeats in TATAA box of UDP-glucuronosyltransferase (UGT)1A1 promoter in archaic hominin, human and non-human primate samples**

To investigate species-specific differences in a microsatellite of UGT1A1 related to variability in plasma bilirubin levels in humans[[1](#_ENREF_1)], we analyzed the A(TA)_n_TAA polymorphism in the 5’ promoter region of UGT1A1 in the sequences of archaic hominins, non-human primates and present-day humans. Sequences of the TATAA box of the promoter of human UGT1A1 (GRCh38 Primary assembly, Chromosome 2q37, position 233,760,234 to 233,760,249) were derived from published sequence data and in the case of the common marmoset its draft assembly (GCA_000004665.1) from the Ensembl database [[2-6](#_ENREF_2)]. As observed by Hall et al [[7](#_ENREF_7)] there was no overlap in copy-number repeats between humans and non-human primates. Archaic hominins had TA-repeat lengths similar to those in present-day humans living outside of Africa.

**Table S1: Variability of TA repeats in TATA box of UDP-glucuronosyltransferase (UGT) 1A1 promoter in archaic hominins, humans and non-human primates**

|  | **A(TA)_n_TAA** | **N** |
| --- | --- | --- |
| **human population[**[**6**](#_ENREF_6)**]** | 5-8 | 13 |
| Africans | 5-8 | 5 |
| Dinka | 6/7 | 1 |
| Mandenka | 5/7 | 1 |
| Mbuti | 7/8 | 1 |
| San | 6/7 | 1 |
| Yoruba | 6/7 | 1 |
| Europeans | 6-7 | 2 |
| French | 6/6 | 1 |
| Sardinians | 6/7 | 1 |
| Asians | 6 | 2 |
| Han | 6/6 | 1 |
| Dai | 6/6 | 1 |
| Australians | 6/6 | 1 |
| Papuan | 6/6 | 1 |
| native Americans | 6-7 | 2 |
| Karitiana | 6/6 | 1 |
| Mixe | 6/7 | 1 |
| **Archaic hominins[**[**4**](#_ENREF_4)**,**[**6**](#_ENREF_6)**]** | 6 | 2 |
| Altai | 6 | 1 |
| Denisova | 6 | 1 |
| **Non-human primates[**[**2**](#_ENREF_2)**,**[**3**](#_ENREF_3)**,**[**5**](#_ENREF_5)**,**[**8**](#_ENREF_8)**]** | 0-4 | 10 |
| chimpanzees | 0, 3-4 | 5 |
| West African | 0, 3-4 | 3 |
| Central African | 3 | 1 |
| East African | 3 | 1 |
| bonobos | 2, 4 | 2 |
| orangutan | 1 | 1 |
| rhesus macaque | 3 | 1 |
| marmoset | 0 | 1 |

1. Borucki K, Weikert C, Fisher E, Jakubiczka S, Luley C, et al. (2009) Haplotypes in the< i> UGT1A1</i> gene and their role as genetic determinants of bilirubin concentration in healthy German volunteers. Clinical biochemistry 42: 1635-1641.

2. Flicek P, Ahmed I, Amode MR, Barrell D, Beal K, et al. (2013) Ensembl 2013. Nucleic Acids Research 41: D48-D55.

3. Locke DP, Hillier LW, Warren WC, Worley KC, Nazareth LV, et al. (2011) Comparative and demographic analysis of orang-utan genomes. Nature 469: 529-533.

4. Meyer M, Kircher M, Gansauge M-T, Li H, Racimo F, et al. (2012) A high-coverage genome sequence from an archaic Denisovan individual. Science 338: 222-226.

5. Prüfer K, Munch K, Hellmann I, Akagi K, Miller JR, et al. (2012) The bonobo genome compared with the chimpanzee and human genomes. Nature.

6. Prüfer K, Racimo F, Patterson N, Jay F, Sankararaman S, et al. (2014) The complete genome sequence of a Neanderthal from the Altai Mountains. Nature 505: 43-49.

7. Hall D, Ybazeta G, Destro-Bisol G, Petzl-Erler M, Di Rienzo A (1999) Variability at the uridine diphosphate glucuronosyltransferase 1A1 promoter in human populations and primates. Pharmacogenetics 9: 591.

8. Gibbs RA, Rogers J, Katze MG, Bumgarner R, Weinstock GM, et al. (2007) Evolutionary and biomedical insights from the rhesus macaque genome. Science 316: 222-234.
