# Supplementary material for: Lineage-Specific Changes in Biomarkers in Great Apes and Humans
Source: PLoS One. 2015 Aug 6;10(8):e0134548. doi: 10.1371/journal.pone.0134548 (PMC4527672; doi:10.1371/journal.pone.0134548)
Supplement: S2 Table — Data derived from [47, 114, 115]. (DOCX) [file pone.0134548.s008.docx]

# Table S2:

**Expression data of genes analyzed in this study**

To compare expression data between humans and chimpanzees and humans and rhesus macaques, we used brain transcriptome data from Babbitt, Fedrigo el al. [[1](#_ENREF_1)], liver transcriptome data from Blekhman, Marioni et al. [[2](#_ENREF_2)] and heart transcriptome data from Dannemann et al. [[3](#_ENREF_3)]

| Gene name | tissue | human vs. chimpanzee |  | human vs. rhesus |  |
| --- | --- | --- | --- | --- | --- |
|  |  | fold-change (log2n) | p-value | fold-change (log2n) | p-value |
| ACACA | liver | -0.22 | 0.93 | -0.6 | 0.08 |
| ACLY | liver | 0.28 | 0.35 | -0.07 | 0.83 |
| ALB | liver | 0.77 | 0.03 | 0.54 | 0.11 |
| BLVRA | liver | 0.28 | 0.39 | 0.34 | 0.48 |
| BLVRB | liver | -0.52 | 0.15 | 0.02 | 0.88 |
| FASN | liver | 0.65 | 0.03 | 0.56 | 0.11 |
| GPD1 | liver | 0.37 | 0.25 | -0.06 | 0.81 |
| GPD2 | liver | 0.37 | 0.35 | -0.42 | 0.06 |
| GRIN1 | brain | 3.19 | 0.06 | 4.03 | 0.02 |
| HMOX1 | liver | -0.22 | 0.55 | 0.18 | 0.59 |
| HMOX2 | liver | -0.26 | 0.38 | -0.38 | 0.05 |
| MBP | brain | 0.49 | 0.46 | 1.9 | 0,000840 |
| ME1 | brain | 0.06 | 0.9 | 1.1 | 0.06 |
| ME1 | liver | 0.2 | 0.53 | 0.01 | 0.94 |
| NPPB | Heart | nondetectable | - | nondetectable | - |
| NRGN | brain | 0.09 | 0.88 | 1.21 | 0.04 |
| UGT1A1 | liver | -0.43 | 0.27 | -0.55 | 0.15 |

1. Babbitt CC, Fedrigo O, Pfefferle AD, Boyle AP, Horvath JE, et al. (2010) Both noncoding and protein-coding RNAs contribute to gene expression evolution in the primate brain. Genome Biology and Evolution 2: 67.

2. Blekhman R, Marioni JC, Zumbo P, Stephens M, Gilad Y (2010) Sex-specific and lineage-specific alternative splicing in primates. Genome Res 20: 180-189.

3. Dannemann M, Prüfer K, Lizano E, Nickel B, Burbano HA, et al. (2012) Transcription factors are targeted by differentially expressed miRNAs in primates. Genome Biology and Evolution 4: 552-564.
